# Derivative processes for modelling metabolic fluxes

**DOI:** 10.1093/bioinformatics/btu069

**Published:** 2014-02-26

**Authors:** Justina Žurauskienė, Paul Kirk, Thomas Thorne, John Pinney, Michael Stumpf

**Affiliations:** Theoretical Systems Biology Group, Centre for Bioinformatics, Imperial College London, South Kensington Campus, London SW7 2AZ, UK

## Abstract

**Motivation:** One of the challenging questions in modelling biological systems is to characterize the functional forms of the processes that control and orchestrate molecular and cellular phenotypes. Recently proposed methods for the analysis of metabolic pathways, for example, dynamic flux estimation, can only provide estimates of the underlying fluxes at discrete time points but fail to capture the complete temporal behaviour. To describe the dynamic variation of the fluxes, we additionally require the assumption of specific functional forms that can capture the temporal behaviour. However, it also remains unclear how to address the noise which might be present in experimentally measured metabolite concentrations.

**Results:** Here we propose a novel approach to modelling metabolic fluxes: derivative processes that are based on multiple-output Gaussian processes (MGPs), which are a flexible non-parametric Bayesian modelling technique. The main advantages that follow from MGPs approach include the natural non-parametric representation of the fluxes and ability to impute the missing data in between the measurements. Our derivative process approach allows us to model changes in metabolite derivative concentrations and to characterize the temporal behaviour of metabolic fluxes from time course data. Because the derivative of a Gaussian process is itself a Gaussian process, we can readily link metabolite concentrations to metabolic fluxes and vice versa. Here we discuss how this can be implemented in an MGP framework and illustrate its application to simple models, including nitrogen metabolism in *Escherichia coli*.

**Availability and implementation:** R code is available from the authors upon request.

**Contact:** j.norkunaite@imperial.ac.uk; m.stumpf@imperial.ac.uk

**Supplementary information:** Supplementary data are available at *Bioinformatics* online.

## 1 INTRODUCTION

It is generally impossible to simultaneously measure the abundance of all the molecular entities making up biological systems. In gene expression assays, for example, we typically measure messenger RNA expression, but not the activity of transcription factors and/or the occupancy of transcription-factor binding sites. Similarly, in metabolomic analyses (Chou and Voit, 2012; Voit, 2013), key metabolites can be measured using, e.g. mass spectrometry or nuclear magnetic resonance quantification, but it is rarely possible to comprehensively quantify the metabolites even within a single pathway. Typically, more interesting than metabolite and enzyme abundance are the fluxes through biochemical reactions and metabolic networks (Orth *et al.*, 2010; Schuster *et al.*, 1999). Fluxes, 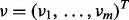, correspond to the rates at which molecules, 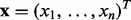, are turned over by the *m* reactions; regulation of fluxes in light of changes in environmental and physiological conditions is also intimately linked to cellular physiology.

Although the fluxes are of central concern, they are hard to measure directly. Estimates for intracellular fluxes can be obtained by tracking products from isotope-labeled (^13^C and ^15^N metabolic flux analysis) metabolites through the metabolic network (Blank and Ebert, 2012; Zamboni, 2011). However, such an approach is restricted to a metabolically steady-state analysis and is not appropriate for capturing dynamical flux variations. Instead, theoretical analysis has often progressed by assuming stationarity of the metabolic processes, which in turn allows for characterizing the sets of steady-state fluxes under a set of suitable assumptions (Klamt and Stelling, 2003; Schwartz and Kanehisa, 2006; Voit and Almeida, 2004). Flux-balance analysis is the most popular example of this strategy, but it becomes questionable once the steady-state assumption can no longer be upheld. Furthermore, as more data on enzyme abundance become available, we should attempt to include such information and the impact on metabolic processes (Colijn *et al.* 2009; Rossell *et al.*, 2013).

Here we provide a new framework that allows us to model metabolic fluxes and their dynamics, and which deals with the missing data problem in metabolic analysis in a flexible and consistent manner. Gaussian processes (GP) belong to the armoury of non–parametric Bayesian methods and have been widely used to describe dynamical processes (Kirk and Stumpf, 2009) and to infer hidden states, e.g. transcription-factor activities (Honkela *et al.*, 2010). In applications to metabolic modelling, parametric approaches can offer potentially incorrect representations of the underlying fluxes (Voit, 2013). The strengths of GP models arise from their non-parametric nature, which enables us to put priors directly on a function rather than on the parameters of a parametric function. With a multiple-output GPs (MGPs), single GP framework can be extended to handle many outputs, enabling us to learn the unknown relationships between metabolic species. In turn, MGPs can be used to infill the sparsely sampled data (Boyle and Frean, 2004). This means that by using MGPs, it is possible to impute the missing data in between the metabolic measurements more efficiently.

Here we develop a more general framework that uses so-called derivative GPs (Solak *et al.*, 2003), which allow us to link metabolite abundance, **x** (or concentrations) and fluxes 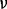. This in turn enables us to also treat time course data on metabolites and monitor the changes that occur in fluxes, e.g. over the course of physiological responses, such as to changes in the environment (Bryant *et al.*, 2013).

## 2 METHODS

### 2.1 GP regression

Gaussian process regression (GPR) can be applied to recover an underlying dynamical process from noisy observations. A GP defines a prior distribution over all possible functions, and to specify a GP, we need expressions for the mean and covariance function that describe the behaviour of the system output over time (Haykin and Moher, 2010). Below we review the standard GPR methodology.

In a typical regression problem, we connect inputs **x** and outputs **z** via functions, 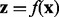, where 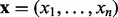 and 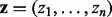 are continuous *n*-dimensional real-valued vectors. The observed values of the dependent variable, **z**, can be related to the independent variables, 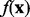 through,

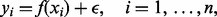

where *ε* is a noise term, which is here assumed to be independent and identically distributed according to a Gaussian distribution, 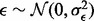. In GPR, we place a GP (Haykin and Moher, 2010; McKay, 1998) prior over the functions 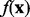, 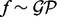, meaning that at any finite number of input points 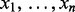 the values 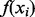 have a multivariate Gaussian distribution with zero mean and covariance function, *K*,

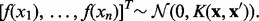



Different functional forms can be chosen for the covariance function (Rasmussen and Williams, 2006), either to simplify computations or to reflect constrains imposed by the data. A flexible and generic choice is to set the covariance function to

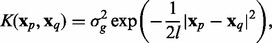

where 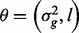 represent a set of unknown hyper-parameters, and 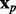 and 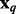 are inputs. Thus, 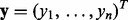 has a multivariate normal distribution with zero mean and covariance matrix 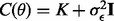, with **I** the identity matrix. The unknown set of hyper-parameters, θ, can be estimated from the data by evaluating the following log-likelihood function,
(1)
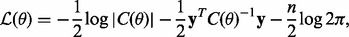

using either a maximum likelihood approach or by sampling from the posterior distribution with Markov chain Monte Carlo methods (Neal, 1997).

For any finite number of input (test) points, 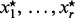, we define the joint prior probability distribution

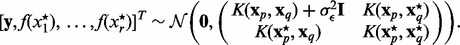



With the GP prior, it is possible to evaluate the posterior distribution over the functions; the values of *f* evaluated at inputs 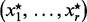 and conditioned on the observations **y** are jointly distributed as (Rasmussen and Williams, 2006),
(2)
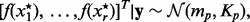

where

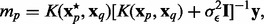

and

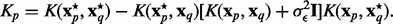



Although Equation (2) defines an appropriate GP posterior, which allows us to make predictions about a single variable **y**, it remains unclear how to deal with several variables simultaneously: if outputs are correlated then the standard GPR framework may fail in providing an adequate description.

### 2.2 Multiple–output GPs

Boyle and Frean (2004) introduced MGPs, where a set of dependent GPs is constructed via multiple-input multiple-output linear filters. This perspective can capture the dependencies between several variables by solving a convolution integral and specifying a suitable covariance function, which in turn includes the cross and auto correlations among related variables. Our construction of derivative processes below builds on MGPs.

Dealing with linear filters is central to signal processing where such filters describe a physical systems that can generate an output signal in response to a given input signal (Haykin and Moher, 2010; Roberts, 2008). Linear filters are characterized by their kernel function (an impulse response) *h*(*t*), and the output *z*(*t*) can be expressed via convolution integral,

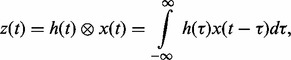

where the symbol ‘
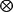
’ denotes the convolution operator. To transmit the signal that has the mathematical properties of a GP, the kernel function, *h*(*t*) must be absolutely integrable, i.e.

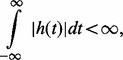



Then if the input *X*(*t*) is specified to be a Gaussian white noise process, the output process, *Z*(*t*), will also be a GP.

Specifying a stable linear time-invariant filter with *M* white noise processes as inputs, 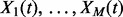, *K* outputs, 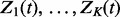 and 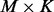 impulse responses results in a dependent GP model (Boyle and Frean, 2005). A multiple-input multiple-output filter can thus be defined as

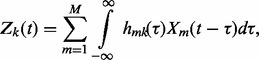

where 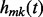 are kernel functions and 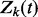 is the *k*th output. As discussed previously, the observed variables might differ from expected variables owing to the measurement noise, and we thus consider
(3)
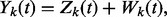

where 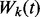 is a Gaussian white noise process with variance 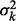.

Multiple-input multiple-output filters are able to capture the relationships between several variables 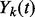; in the model, these kind of dependencies are build in via shared input noise sources that enable the specification of valid covariance functions. For the sake of simplicity, let the impulse response be a Gaussian kernel, 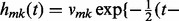 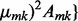. Then evaluating the convolution integral leads to the following covariance function,
(4)
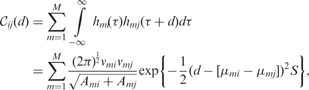

where 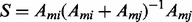 and 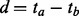 is the temporal separation between two input points, (see Boyle and Frean (2004) appendix for derivation and generalization to multidimensions). Constructing intermediate matrices *C_ij_* permits the definition of a positive definite symmetric covariance matrix **C** between *K* variables,

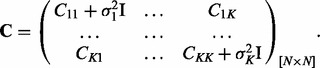



Here 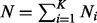 is total number of observations, and *N_i_* the number of observations of variable *i*. Having defined the covariance matrix, we can use the log-likelihood, which has the form (1) for the inference of the hyper-parameters 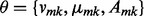. Again, following Bayesian framework, we can use the results from the GPR section to evaluate the joint predictive distribution (2) for all outputs. Alternatively, for a particular variable *i*, predictions can be made using the appropriate marginal distribution, which is Gaussian, with mean 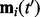 and variance 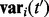, given by
(5)
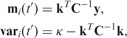

where

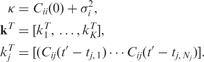



### 2.3 Derivative processes

For a GP that is derived through a linear filter, 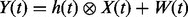, where *X*(*t*) is a white noise GP, *h*(*t*) is a kernel function and *W*(*t*) is an additive noise, it is easy to formulate the expression of a derivative process. Taking a derivative of *Y* with respect to *t*, it is possible to obtain a new process *U* that is also a GP (Boyle, 2007),

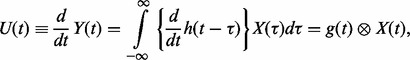



Thus, it is possible to construct the derivative process by convolving a white noise GP *X*(*t*) with a derivative kernel function *g*(*t*). This definition enables us to consider derivative processes and the corresponding original processes as a collection of dependent GPs. This is true because the derivative processes and the original processes are derived from exactly the same input, *X*(*t*).

To construct a dependent model for several related variables 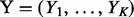 and their derivatives 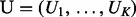, it is necessary to define a suitable covariance structure, which in principal arises from the initial covariance function (4). For example, for a set of four dependent outputs (two original and two derivative processes), the following equations can be applied to compute the covariances (Girard, 2004; Kirk, 2011; Solak *et al.*, 2003),

- Autocovariance function of derivative process *U_i_*

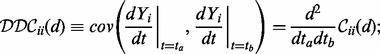

- Cross-covariance function between two derivative processes *U_i_* and *U_j_*

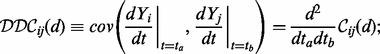

- Covariance between original process *Y_i_* and corresponding derivative process *U_i_*

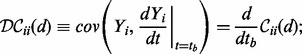

- Covariance between original process *Y_i_* and derivative process *U_j_*

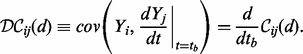

Let **R** denote a block matrix,

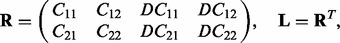

which describes the correlations between observations 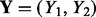 and their ‘function’ values 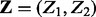, and corresponding derivative variables 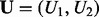 evaluated at any finite number of test points 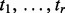. In a similar manner, let **H** denote

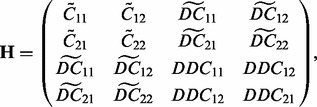

where the 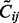 matrices contain the correlations between functions *Z*_1_ and *Z*_2_ evaluated at a finite set of test points 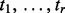; 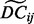 the correlations between functions 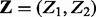 and derivative variables 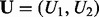 evaluated at the same test points; and finally, *DDC_ij_* consists of auto/cross-correlations between derivative variables *U*_1_ and *U*_2_. The matrices 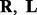 and **H** are building components of the overall covariance matrix **K**, which is symmetric and positive definite,

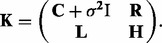



At a finite number of input points 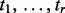, the matrix 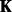 allows us to place a joint prior over observations **Y**, functions **Z** and derivatives **U**,

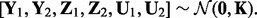



Evaluating a GP posterior
(6)
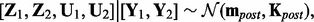

where

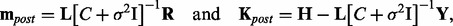

enables us to make joint predictions for the original and derivative processes simultaneously. Alternatively, if there is no need to sample from the posterior process, we can use marginal Gaussian distributions to make predictions for individual output. The marginal distributions for output *i* and its derivative process at any input point 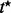,
(7)
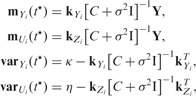

where 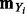 is the mean of the original process, 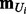 the mean of the derivative process, 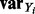 the variance of the original process and 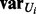 the variance of the derivative process, and furthermore

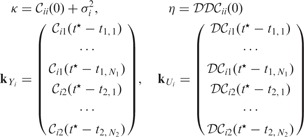



Equations (6) and (7) can easily be extended to make predictions about any number of variables.

## 3 APPLICATIONS AND RESULTS

To demonstrate the performance of derivative processes, we consider two simulation examples—a system of two oscillating signals and a simple model of linear metabolic pathway—before turning to a more complicated metabolic process and, finally, some real metabolic network data. The derivative processes can be used to address the flux estimation problem from time course data. Here GPs describe the dynamics of metabolites, and the corresponding derivative processes capture the functional forms of the associated fluxes. Below, all examples were implemented using the free statistical computing platform *R* www.r-project.org.

### 3.1 Oscillating signals

**Fig. 1.**
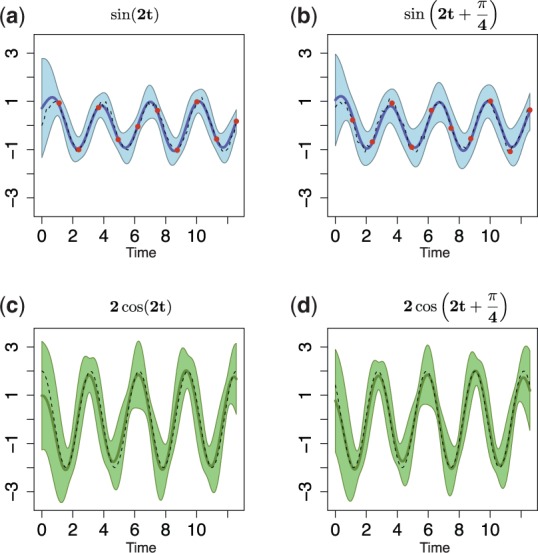
Predictions with MGPs model for two oscillating signals. (**a** and **b**) Dashed lines represent true behaviour of noiseless 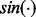 trajectories; dots correspond to the noisy observations for both signals (data); solid lines are the mean behaviour of the MGPs model (predictions with original GPs); light areas correspond to two standard deviations at each prediction point. (**c** and **d**) Dashed lines represent true behaviour of noiseless 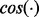 trajectories; solid lines show the mean behaviour of the MGPs model (predictions with derivative processes); light areas correspond to two standard deviations at each prediction point

A simple oscillating signal can be expressed as 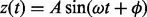, where *A* is the amplitude, 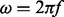 the angular frequency and 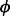 the phase angle. This is a particularly useful example because it is easy to evaluate the performance of derivative processes, as the derivative signals have a known analytic form. We consider a simple system that consists of two oscillating signals, 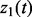 and 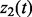,

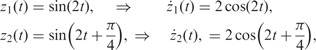

with 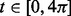. To model real experimental measurements, we add random noise to the simulated trajectories, 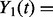 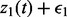, 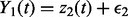, where 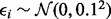; we have observations of both signals at regular time intervals, 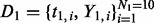 and 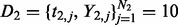. To build a single model that captures the relationship between the two signals, we apply the dependent GP framework (3) (K = 2) on a combined dataset 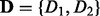; each signal can be expressed as a superposition of three GPs—two of which are constructed via convolution between a noise source and a Gaussian kernel, and the third one is an additive noise. We set parameters *A_i_* of each Gaussian kernel to be 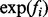 and noise levels to 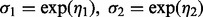, leading to a set of hyper-parameters 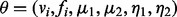, 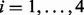. To build the model the following priors are chosen: 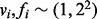, 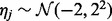 and 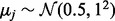, 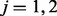; the maximum a posteriori (MAP) estimate 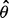 is determined using a multistarting Nelder–Mead optimization algorithm (Nelder and Mead, 1965). Dependent GP posteriors (6) allow us to make joint predictions about both signals and their derivative processes at any finite number of input points, and the resulting posterior processes are summarized in Figure 1. From these posterior processes, it can be seen that the mean behaviour of our model agrees with trajectories of underlying noiseless signals, and to make predictions about derivative processes, it is enough to consider only samples from the original sinusoidal trajectories.

### 3.2 Linear pathway

Next we consider a linear metabolic pathway with two regulatory signals (see Goel *et al.* (2008) Supplementary Material for details), which is summarized in Figure 3a. Here the flow from *x*_1_ to *x*_2_ is negatively regulated by metabolite *x*_3_, and *x*_3_ increases the transformation of *x*_2_ into *x*_3_. A set of ordinary differential equations (ODEs) can be used to describe the dynamics of these two metabolites, *x*_2_ and *x*_3_ (*x*_1_ is the constant external input),
(8)
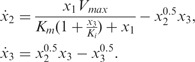



**Fig. 2.**
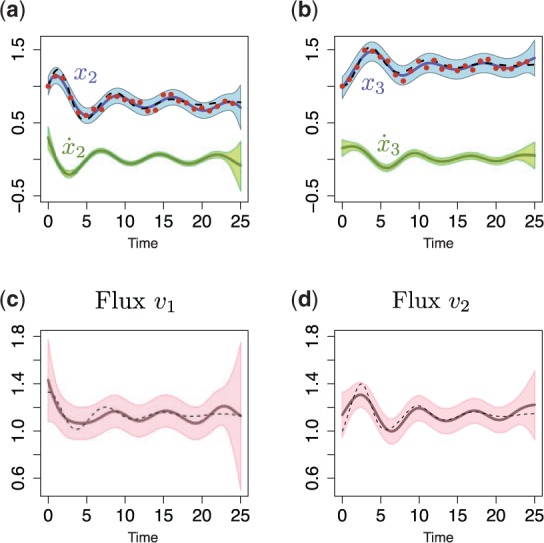
Predictions with MGPs model for linear metabolic pathway. (**a** and **b**) Dashed lines represent a simulated *x*_2_ and *x*_3_ trajectories from ODE model; dots correspond to the sparse noisy observations for *x*_2_ and *x*_3_ (data); solid blue/green lines are the mean behaviour of the MGPs model (blue, predictions with original GPs; green, predictions with derivative process); light areas correspond to two standard deviations at each prediction point. (**c** and **d**) Dark lines are predicted fluxes, light areas correspond to the confidence region, and dashed lines represent true behaviour of noise-free fluxes *v*_1_ and *v*_2_ (calculated from ODE system)

To apply the derivative process approach, we simulate the ODE model with the following parameter values 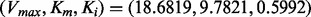 and initial conditions 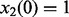, 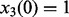. In this model, the concentration of *x*_1_ is assumed to be constant and equal to 2. The dataset consists of selected points from simulated trajectories with added Gaussian noise 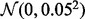. Again we combine the ‘noisy’ measurements, and fit the dependent GP model to make predictions about the original trajectories and their derivatives. To obtain a functional expressions for fluxes *v*_1_ and *v*_2_ we need to estimate a dynamical variations of metabolic, 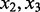, derivatives. The derivative processes provide the predictions for the left side of Equation (8) at any finite number of time points, whereas the original GPs describe the solution on the same ODE (8). This enables us to link the metabolite measurements to metabolic fluxes. Figure 2 illustrates the predictions with posterior processes, where solid blue lines correspond to the mean behaviour of the model, dashed lines to the original *x*_2_ and *x*_3_ trajectories and solid green lines to their derivatives. In addition, if we assume that we are able to measure flux 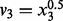, we can obtain the functional expressions for fluxes *v*_1_ and *v*_2_ that are summarized in Figure 2c and d. The dark pink lines illustrate predicted fluxes from noisy metabolite measurements, dashed lines are real fluxes (calculated from ODEs (8)) and light pink area corresponds to the confidence region.

**Fig. 3.**
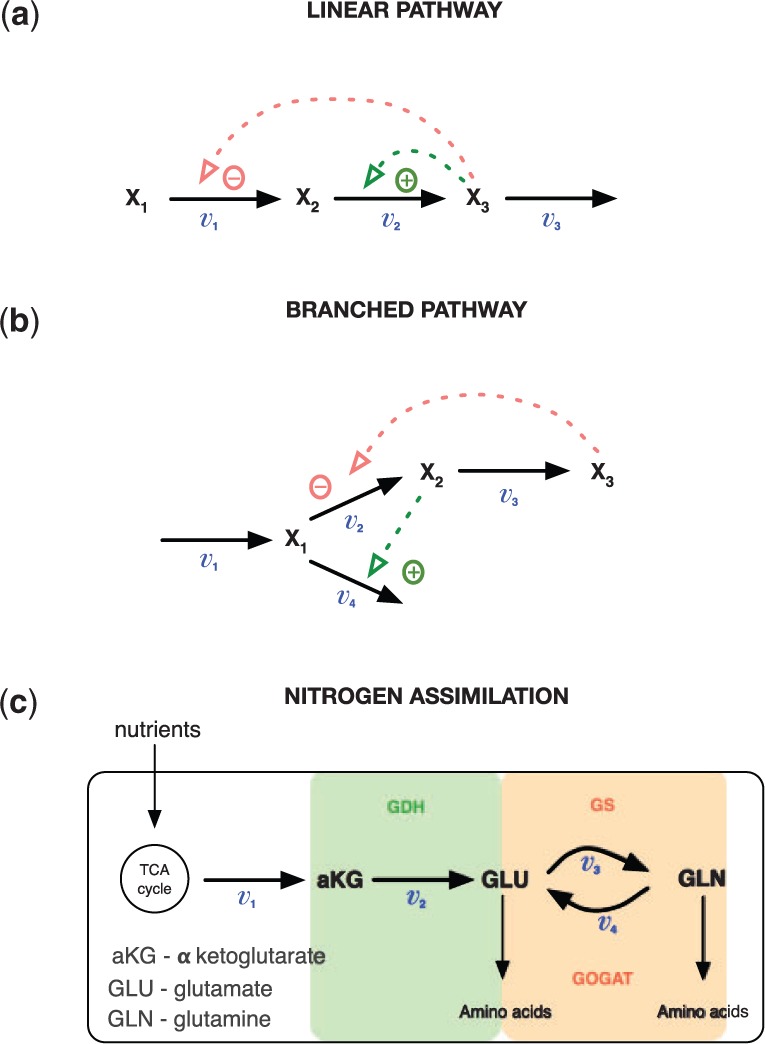
Pathway information. (**a**) A simple linear metabolic pathway; red and green dashed lines correspond to the inhibition and activation signals. (**b**) Illustrates a branched pathway with positive (green) and negative (red) regulatory signals. (**c**) Illustrates a metabolic pathway in *E.coli*, here *v_i_*, 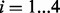 denote the fluxes; 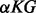, *GLU* and *GLN* correspond to the metabolites; TCA is a short notation for the citrate cycle in *E.coli*

### 3.3 Branched pathway

We now turn to an example of metabolic pathway that was originally proposed by Voit (2013) (see *Example of actual characterization*); Figure 3b illustrates a schematic representation of a branched pathway with two regulatory responses, where *x*_3_ inhibits the conversions of *x*_1_ into *x*_2_, and *x*_2_ positively regulates reaction *v*_4_. The following ODE model describes the dynamics of the metabolites that are involved in this pathway,
(9)
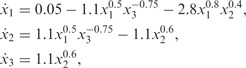

where 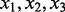 denote the metabolites. For a given pathway (Fig. 3b), the change in metabolite concentration can be described by the differences between incoming and outgoing fluxes. For this reason, we are able to obtain the following expressions for fluxes 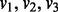 and *v*_4_,
(10)
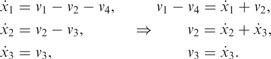



These expressions define a system of linear equations that is underdetermined, as we have more fluxes to estimate than available equations, and it cannot be solved using standard Gaussian elimination techniques. For this reason, additional information is required to uniquely determine fluxes *v*_1_ and *v*_4_. In this example, we will focus only on estimation of fluxes *v*_2_ and *v*_3_ from available data rather than try to address a uniqueness problem of *v*_1_ and *v*_4_.

**Fig. 4.**
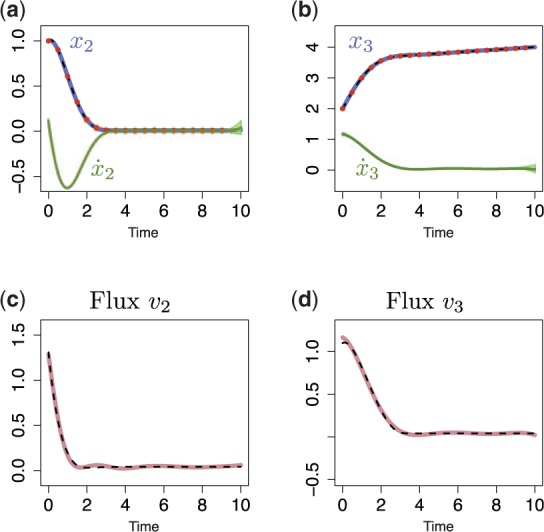
Predictions with MGP model for a branched metabolic pathway. (**a** and **b**) Dashed lines represent simulated *x*_2_ and *x*_3_ trajectories from the ODE model; red dots correspond to the sparse observations for *x*_2_ and *x*_3_ (data); solid lines are the mean behaviour of the MGPs model (blue, predictions with original GPs; green, predictions with derivative process); light areas correspond to two standard deviations at each prediction point. (**c** and **d**) Dark lines are predicted fluxes; dashed lines represent true behaviour of fluxes *v*_2_ and *v*_3_ (calculated from the ODE system)

The above ODE model enables us to generate simulated time course data using the initial conditions 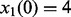, 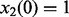 and 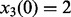. Next, we apply the dependent GP framework (3) (K = 2) on the combined dataset 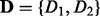, where 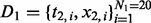 and 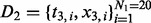 contains the measurements of metabolites *x*_2_ and *x*_3_ with added random Gaussian noise 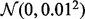 (we chose a low noise level so that predictions with derivative processes could be easily compared with the original fluxes in the example in Voit (2013). For a set of model hyper-parameters 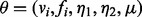, 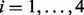 we use the following priors, 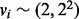, 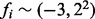, 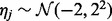, 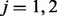 and 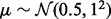, and calculate the MAP estimate 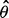 as before. Figure 4 illustrates the predictions with posterior processes using Equation (7); (a and b) graphs summarize metabolite data. The dark blue lines correspond to the mean behaviour of the original GPs and agree well with simulated *x*_2_ and *x*_3_ dynamics; the green lines describe the derivatives of the same metabolites and can be understood as a slope estimates. In Figure 4c and d, dark pink lines illustrate the predicted metabolic fluxes *v*_2_ and *v*_3_ under consideration of pathway Figure 3b. From ODE model (9), we can calculate original fluxes over the time (in real situations this would not be possible). Figure 4c and d shows a good agreement between predicted and original fluxes.

### 3.4 *Escherichia coli* nitrogen assimilation

**Fig. 5.**
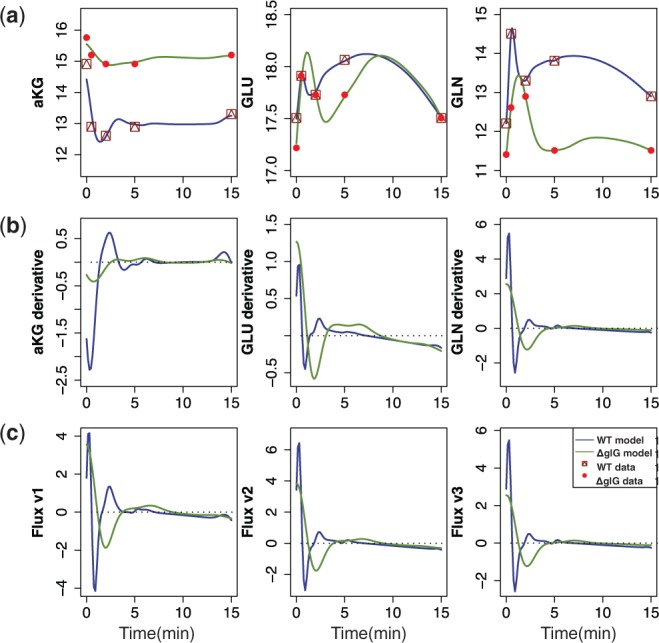
Predictions with MGPs model for *E. coli* (WT and ΔglnG). (**a**) The symbols indicate experimentally measured concentrations of 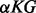, *GLU* and *GLN* metabolites (dots for WT, squares for ΔglnG). Solid lines correspond to the mean behaviour of dependent GPs model. (**b**) Predicted derivative behaviour for 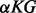, *GLU* and *GLN* metabolites, where solid lines correspond to the mean behaviour of dependent derivative processes. (**c**) Predicted fluxes *v*_1_, *v*_2_ and *v*_3_ for convenience, dotted line illustrates horizontal 0-axis

Finally, we apply our technique to the experimental data from *E. coli*, where we have measurements of the abundance of several key metabolites involved in nitrogen assimilation. Nitrogen is one of the key chemical elements that acts as a nutrient for the cells; ammonium is a preferred source of nitrogen for *E. coli* growth (Schumacher *et al.*, 2013; van Heeswijk *et al.*, 2013). In *E. coli*, ammonium can be absorbed via two pathways: glutamate dehydrogenase (GDH) that operates during cell growth in ammonium-rich environments, and glutamine synthetase-glutamate synthase (GS-GOGAT) that operates during cell growth in low-ammonium conditions (van Heeswijk *et al.*, 2013). Here, we are focussing on experimental conditions, where after a period of nitrogen starvation, the bacterial cultures are spiked with ammonium (Schumacher *et al.*, 2013); Figure 5a shows experimentally obtained measurements for *α-ketoglutarade* (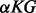), *glutamate* (*GLU*) and *glutamine* (GLN) metabolites over the time after ammonium spike; red dots correspond to a wild-type (WT) *E. coli* metabolic measurements, and in squares—isogenic glnG deletion (ΔglnG) measurements. Below we focus on the pathway summarized in Figure 3c, which includes both GDH and GS-GOGAT. For modelling purposes, we assume that fluxes *v*_3_ and *v*_4_ can be summarized by the overall flux *v*_3_ that describes the flow from GLU to GLN, as there is not enough information to discriminate between them. From the pathway, we can construct a system of linear equations that describe the dependencies between fluxes and metabolites,
(11)
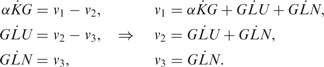



We fit a dependent GP model (3) (K = 3) to the WT data and then to ΔglnG data (collected from a strain where glnG is absent). In the model, 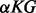 is expressed as a sum of three GPs: the first GP describes 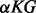, the second expresses the relationship between 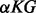 and *GLU* and the third one describes additive noise; *GLN* is modelled similarly. However, *GLU* is modelled as the sum of four GPs, where the first three describe *GLU*; the dependence between *GLU* and 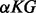; the dependence between *GLU* and *GLN*; and the fourth is an additive noise. Choosing kernel functions to be Gaussian 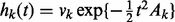, we obtain the MAP estimate for all hyper-parameters (17 in total). The predictions with posterior process (7) are summarized in Figure 5, where solid blue lines describe predictions with dependent GP models for WT *E. coli*, and green lines for ΔglnG. Using the relationship (11), we can estimate fluxes *v*_1_, *v*_2_ and *v*_3_ (Fig. 5c).

To evaluate our predictions, we can compare flux *v*_3_ and GS protein levels in WT and ΔglnG *E. coli* (see Supplementary Fig. S1). In *E. coli*, glnG encodes the transcription factor, NtrC (nitrogen regulator) that controls GS expression levels, and in its active form, GS catalyses glutamine synthesis (van Heeswijk *et al.*, 2013). Experimentally, it was observed that in ΔglnG case protein, GS levels were significantly lower compared with the GS levels in WT *E. coli* (see Supplementary Fig. S1C and D). Because there is less enzyme available to catalyse the reaction in ΔglnG, the flux *v*_3_ in the mutant will be noticeably reduced compared with the WT flux *v*_3_ (see Supplementary Fig. S1A and B).

## 4 DISCUSSION AND CONCLUSIONS

Flux estimation has become central to many analyses into the metabolic processes and mechanisms. Typically, the estimates for a set of fluxes are obtained in a point-wise manner at discrete time points. It is clear that this fails to capture the temporal behaviour of the fluxes and additional consideration of parametric models is compulsory to fully explain the fluxes; further, this approach is susceptible to noise that is present in experimentally measured metabolite data.

Here we have addressed these problems and proposed a novel non-parametric Bayesian approach to modelling metabolic fluxes. This is based on MGPs that enable the construction of derivative processes. Because the derivative processes and original processes share the same input source, we can complement the dependent GP model and make joint predictions about original and derivative processes at any finite number of input points. Such derivative processes can be applied to characterize the temporal behaviour of metabolic fluxes from time course data—without having to make reference, e.g. transcriptomic data, to explain temporal variation—and here we have demonstrated the applicability on simple models and a real-world example.

GPs, including our approach, propagate uncertainty in line with the assumed covariance structures. This can lead to large confidence intervals, especially if the dependencies among different observations are not considered explicitly. With increasing number of metabolic species within the pathway, the derivative process approach might become computationally costly due to the inference of a large number of hyper-parameters and a matrix inversion step; however, this limitation potentially might be addressed by considering a sparse approximation for the full covariance matrix of all metabolic species (Alvarez and Lawrence, 2009). These can in principle deal with genome-level data.

## Supplementary Material

Supplementary Data
