# Supplementary material for: Derivative processes for modelling metabolic fluxes
Source: Bioinformatics. 2014 Feb 26;30(13):1892–8. doi: 10.1093/bioinformatics/btu069 (PMC4071196; doi:10.1093/bioinformatics/btu069)
Supplement: Supplementary Data [file supp_30_13_1892__index.html]

Derivative Processes for Modelling Metabolic Fluxes — Derivative processes for modelling metabolic fluxes — Derivative processes for modelling metabolic fluxes — Supplementary Data 

# Derivative processes for modelling metabolic fluxes

## Supplementary Data

files

**Files in this Data Supplement:**

- Supplementary Data - pdf file
