# Supplementary material for: Derivative processes for modelling metabolic fluxes
Source: Bioinformatics. 2014 Feb 26;30(13):1892–8. doi: 10.1093/bioinformatics/btu069 (PMC4071196; doi:10.1093/bioinformatics/btu069)
Supplement: Supplementary Data [file supp_btu069_supp_fig.pdf]

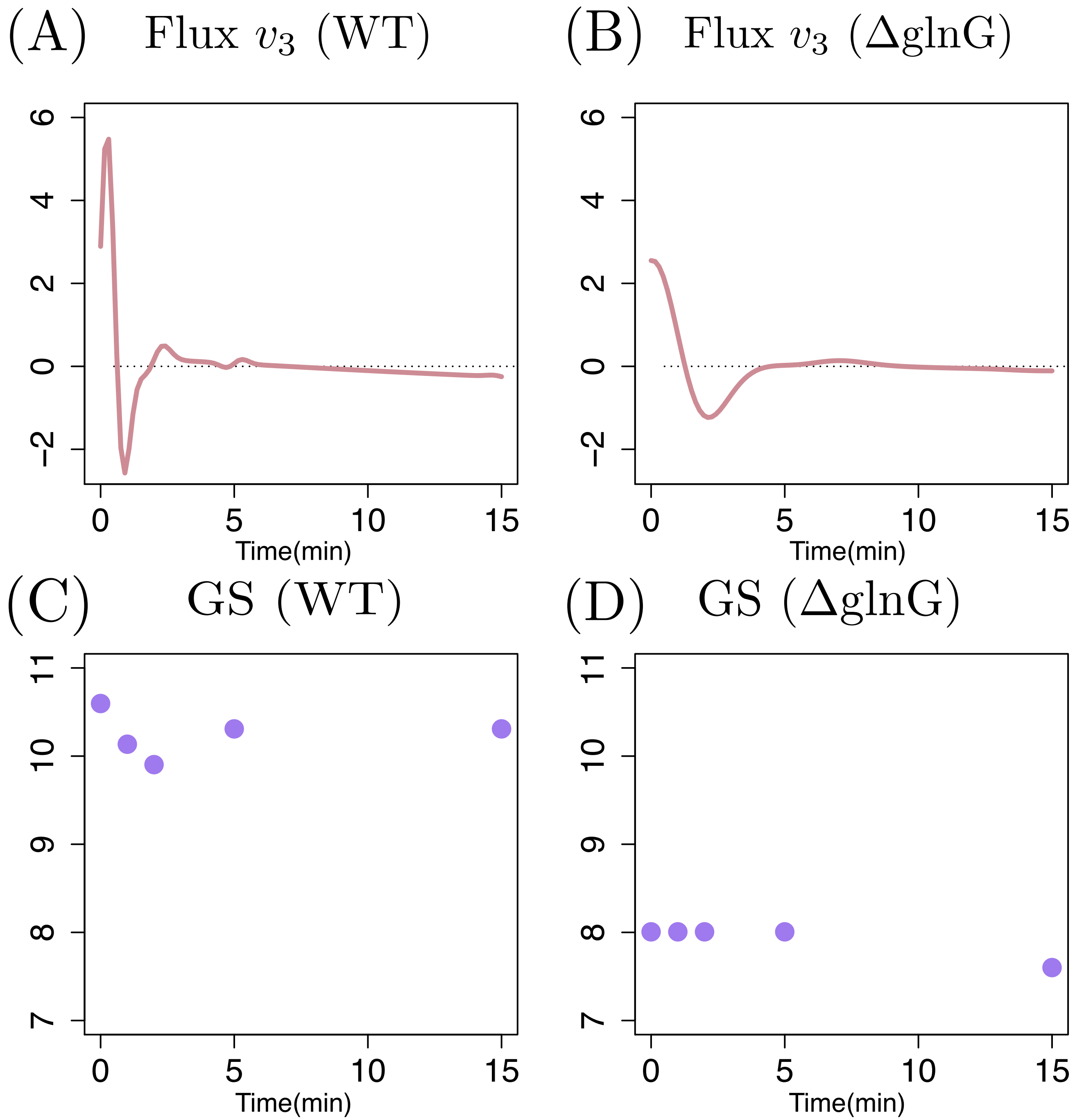

Fig. S1. Flux and glutamine synthetase (GS) comparison.
(*A*) Predicted flux v_3 in wild type *E. Coli*; dotted line illustrates the horizontal 0-axis.
(*B*) Predicted flux v_3 in ΔglnG *E. Coli*; dotted line illustrates the horizontal 0-axis.
(*C*) Measurements of active form (un-adenylylated form) GS protein in wilde type *E. Coli*.
(*D*) Measurements of active form (un-adenylylated form) GS protein in ΔglnG *E. Coli*.
